# CIRSE Standards of Practice on Carotid Artery Stenting

**DOI:** 10.1007/s00270-024-03707-y

**Published:** 2024-04-29

**Authors:** Stavros Spiliopoulos, Raphaël Blanc, Roberto Gandini, Stefan Müller-Hülsbeck, Wolfgang Reith, Ornella Moschovaki-Zeiger

**Affiliations:** 1 Interventional Radiology Unit, 2nd Department of Radiology, National and Kapodistrian University of Athens, “Attikon” University General Hospital, Athens, Greece; 2 Department of Interventional Neuroradiology, Foundation Rothschild Hospital, Paris, France; 3 Diagnostic and Interventional Radiology/Neuroradiology, University of Rome “Tor Vergata”, Rome, Italy; 4 Diagnostic and Interventional Radiology/Neuroradiology, DIAKO Hospital, Flensburg, Germany; 5 Department of Neuroradiology, Saarland University, Homberg, Germany

**Keywords:** Carotid artery, Stenting, Endovascular treatment

## Abstract

**Background:**

Carotid artery stenting has been used effectively to treat internal carotid artery stenosis since 1989 (Mathias et al. in World J Surg. 25(3):328-34, 2001), with refined and expanded techniques and tools presently delivering outstanding results in percutaneous endoluminal treatment of carotid artery stenosis.

**Purpose:**

This CIRSE Standards of Practice document is directed at interventional radiologists and details the guidelines for carotid artery stenting, as well as the different implementation techniques. In addition to updating all previously published material on the different clinical indications, it will provide all technical details reflective of European practice for carotid artery stenting. CIRSE Standards of Practice documents do not aim to implement a standard of clinical patient care, but rather to provide a realistic strategy and best practices for the execution of this procedure.

**Methods:**

The writing group, which was established by the CIRSE Standards of Practice Committee, consisted of five clinicians with internationally recognised expertise in carotid artery stenting procedures. The writing group reviewed existing literature on carotid artery stenting procedures, performing a pragmatic evidence search using PubMed to select relevant publications in the English language from 2006 to 2022.

**Results:**

Carotid artery stenting has an established role in the management of internal carotid artery stenosis; this Standards of Practice document provides up-to-date recommendations for its safe performance.

## Introduction

The CIRSE Standards of Practice Committee established a writing group that was tasked with producing up-to-date recommendations for performing carotid artery stenting (CAS), taking into account data on novel techniques, devices, and recent outcomes that have emerged over the last decade. For the purpose of clarification, it is emphasised that the topic of dissection treatment will not be analysed within this Standards of Practice document. It is important to clarify that this SOP paper is not intended to initiate a discourse comparing CAS to carotid endarterectomy (CEA); rather, the primary goal is to establish a comprehensive set of guidelines for state-of-the-art CAS procedures. As CIRSE Standards of Practice documents are not clinical practice guidelines or systematic reviews of the literature, this document is not intended to impose a standard of clinical patient care but recommend a reasonable approach to and best practices for performing CAS [1].

## Methods

The writing group, which was established by the CIRSE Standards of Practice Committee, consisted of five clinicians with internationally recognised expertise in CAS procedures. The writing group reviewed existing literature on CAS procedures, performing a pragmatic evidence search using PubMed to select relevant publications in the English language from 2006 to 2022.

## Background

Around 10–15% of all episodes of ischaemic stroke and transient ischaemic attacks (TIA) may be attributed to atherosclerotic carotid artery disease, whilst the majority of carotid revascularisation interventions (75%) are done on asymptomatic patients for primary prevention of an ischaemic stroke event [2].

In 1994, carotid artery stenting was presented as an alternative to carotid endarterectomy for the treatment of individuals who had been assessed to be candidates for carotid revascularisation [3].

The carotid bifurcation is the most prevalent site for atherosclerotic carotid stenosis to develop, occasionally affecting the distal common and/or the proximal internal carotid arteries [2].

Indications for CAS and its general applicability have considerably expanded as a result of rapid technological developments in both the stents themselves and distal protective devices.

### Indications, Contraindications, and Patient Selection

Carotid artery stenosis is considered symptomatic when correlated with findings like focal neurologic symptoms produced by ipsilateral transient ischaemic attacks, amaurosis fugax, or an ischaemic stroke in the carotid artery territory during the preceding six months. According to the NASCET criteria (North American Symptomatic Carotid Endarterectomy Trial [4]), a high-grade stenosis is characterised as a ≥ 70% stenosis (equivalent to a ≥ 85% stenosis according to ECST criteria – European Carotid Surgery Trial) [2].

### Indications

Symptomatic patients:

1. With high-grade stenoses:
  - Critical tandem lesion;
  - Radiation-induced;
  - After carotid endarterectomy (restenosis);
  - Due to dissection;
  - As a result of fibromuscular dysplasia;
  - Related to Takayasu arteritis.
2. Presenting with pseudoaneurysms.
3. That have opted for CAS as their preferred treatment over CEA.
4. With signs of global hypoperfusion, according to a specialised stroke neurologist [5].
5. With presence of carotid web (although evidence is currently limited and supported primarily by limited case series) [6, 7].

Asymptomatic patients:

1. Of a younger age with a progressive asymptomatic lesion (> 70% stenosis) with or without evidence of cerebral infarction on brain computed tomography (CT) scans [5].
2. With bilateral 79–90% asymptomatic carotid stenosis (with a combined percentage of 160%).
3. With unilateral 70–90% stenosis with contralateral occlusion [8].
4. Who will undergo coronary artery bypass graft and have a previous history of stroke/TIA.

### Contraindications

Absolute contraindications

1. A high-grade stenosis that would be inaccessible using endovascular techniques, due to unfavourable aortic arch anatomy, such as type 3 arch or extensive calcification [5, 9].
2. In a non-acute setting, a high-grade carotid stenosis accompanied by intraluminal thrombus [5].

Relative contraindications:

1. Patients with a high-grade stenosis, in the presence of proximal embolic sources, such as atrial fibrillation, patent foramen ovale, mechanical prosthetic heart valves or other potential embolic causes [5].
  (The implementation of bridging strategies, both pre- and post-procedure, in patients receiving anticoagulant therapy should be undertaken in consultation with their cardiovascular specialist.)
2. Patients exhibiting a high-grade stenosis, and for whom endovascular interventions are precluded due to irreversible coagulopathy.
  (The procedure might need to be postponed until the bleeding risk has been minimised) [10].
3. Patients presenting with high-grade carotid stenosis concomitant with a pre-existing intracranial haemorrhage.
4. Patients with a symptomatic high-grade stenosis with an accompanying cerebral vascular malformation poses a challenging clinical setting, as there is currently a lack of data available for guiding treatment decision [5].

Concerning points 1, 2, and 3, these are viewed as relative contraindications, given that the decision-making process for managing thrombotic and bleeding risks should be tailored to each patient, considering the severity of the symptomatology associated with the carotid stenosis.

## Patient Preparation

### Pre-procedural Work-Up

#### Informed Consent

The patient should be provided with reliable clinical data on the risks and benefits of CAS, CEA and pharmacological therapy in order to be able to make informed treatment choices, with the advice and recommendations of the treating physician [5].

#### Technical Requirements

- The CAS procedure requires a single-plane angiography suite, and ideally, a bi-plane angiography suite would be even more advantageous.
- The suite should provide adequate space for the set-up of monitoring equipment, whilst also facilitating personnel movement without compromising the sterile field.
- Equipment for cardiopulmonary resuscitation needs to be included amongst the necessary tools required for peri-procedural physiological monitoring.
- A beneficial tool for guidance is road-mapping (real-time digital subtraction) with the ability to calibrate and measure, as it paves the way for properly sized carotid stents and proper protective device placement.
- It is also important that the process be carried out in hospitals or other facilities that have neuroresuscitation experts readily available [5].

#### Pre-operative Preparation

- Complete neurologic evaluation
  o Create a reference point for the purpose of future comparison
- Anticoagulation
  o Initiate dual antiplatelet therapy (DAPT) escalation by administering aspirin (100 mg) and clopidogrel (75 mg) for 5–7 days before the scheduled procedure, following a platelet function test; or
  o A single loading dose clopidogrel 300 mg 6 hours prior to procedure, in urgent situations [8].
- Renal function evaluation and preparation
  o Routine laboratory tests (serum creatinine and urea) should be obtained.
  o Reduction of renal injury, particularly in high-risk patients (diabetes, renal impairment), can be accomplished with fluid administration. Intravenous (IV) administration of saline 1.0–1.5 ml/kg/h for at least six hours before and after the procedure is commonly recommended [5, 11].

### Pre-procedural Imaging

Non-invasive imaging modalities, including duplex ultrasound (US), CT angiography (CTA) and MR angiography (MRA) have largely superseded traditional endovascular cerebral angiography for the detection of carotid artery stenosis, due to the low procedural risks, low cost and shorter examination times [12].

Pre-procedural CTA or MRA imaging as an add-on to Doppler ultrasound (DUS) is commonly performed, and is particularly useful.

The degree of the stenosis is calculated by either the NASCET criteria, that calculates the ratio of the stenosis to the normal lumen distal to the stenosis, or the ECST criteria, which characterises the degree of the stenosis in relation to the estimated normal lumen at the same level [13].

*Duplex ultrasonography* utilises blood velocity in addition to B-mode greyscale to detect the existence and degree of stenosis, as well as evaluation of plaque composition and characteristics.

A high-grade stenosis (> 70%) is defined as a peak systolic velocity greater than 230 cm/sec, an end diastolic velocity greater than 100 cm/sec, an internal carotid to common carotid artery ratio greater than 4.0, and the presence of visible plaque, according to the Society of Radiologists in Ultrasound (SRU) [14].

*CT angiography*. The aortic arch and the circle of Willis should be depicted for evaluation of the exact anatomy, tortuosity, the degree of the carotid artery stenosis, and the identification of significant tandem stenoses.

*Magnetic resonance angiography* (MRA) can be conducted without the administration of contrast medium, particularly for patients with impaired kidney function. A drawback lies in the ineligibility of patients with non-MR-compatible pacemakers and implants making them unsuitable candidates for MRA. Additionally, the accuracy of identification of vessel wall calcifications is less reliable [15].

*Digital subtraction angiography* (DSA) was the diagnostic modality of choice in the past; however, unless the results of non-invasive investigations are inconclusive and angiography is part of a therapeutic intervention, clinical guidelines advise against performing diagnostic angiography with selective carotid artery catheterisation due to the increased risk of stroke and distal embolisation from wire and catheter manipulation in the arch and its branches in addition to access site complications [16].

### Timing of CAS (in Symptomatic Patients)

- Intervention within two weeks of TIA onset is most beneficial for symptomatic patients with ≥ 50% stenosis (NASCET) [2, 17, 18].
- For patients who have received intravenous thrombolysis for acute ischaemic stroke with 50–90% carotid stenosis, CAS should only be considered six days post-thrombolysis to minimise post-interventional haemorrhage.
- For patients facing neurological instability (defined as those who have suffered a disabling stroke with a modified Rankin score > 3, or altered consciousness/drowsiness,) and 50–99% carotid stenosis, the recommendation is to postpone CAS (or CEA) until there is evidence of neurological improvement [8, 19, 20].

### Endovascular Devices

Self-expanding stents are recommended for CAS, especially in the proximal carotid artery. Balloon-expandable stents may be employed in the more distal internal carotid segments; however, their relative inflexibility may warrant consideration for the previous option.

Based on the scale of the self-expanding stent’s free-cell area, the devices may be categorised as either open-cell stents (OCS), closed-cell stents (CCS) or the recently introduced dual-layer stents.

OCS bend and flex to conform to the shape of the vessel into which they are being placed, making them a good choice for navigating tortuous arteries, without shortening upon deployment. However, due to a weaker scaffold and decreased coverage of the lesion potentially resulting in plaque protrusion, there is an increased risk of embolization and in-stent restenosis.

CCS have a more effective support and framework, due to their dense structure; however, due to their design, the flexibility to adapt to a curved vessel is limited and might cause kinking or damage. Dual-layer stents or micro-mesh stents are a combination of the adaptability of OCS with the dense weaving of the CCS, designed according to a technology that decreases free-cell area and increases support [21]. It is thought, however, that dual-layer stents exhibit an elevated incidence of early thrombosis, with a degree of suspicion surrounding potential higher rates of restenosis, although several studies have been reporting contradictory findings.

Low-profile, over-the-wire or rapid-exchange balloon catheters can be used for pre- and post-dilation (typically 2–4 mm in diameter for pre-dilation, 4–6 mm for post-dilation, up to 4 cm in length) [22].

## Procedural Details and Endovascular Techniques

### Access

Ultrasound-guided local anaesthesia application at the anterior arterial wall and arterial puncture is advised for all access types, to increase first-pass success rates and reduce the level of access-related pain and complications [23, 24].

#### Transfemoral

Whilst transfemoral access, typically obtained by the common femoral artery (CFA), has historically been the predominant access site, there has been a notable shift in recent years to alternative routes, such as transradial access. Standard 19-G needles or a 21-G micro-puncture set can be used [8].

#### Radial and Brachial

The use of radial or, less frequently, brachial access is increasingly prevalent, particularly in cases of significant lower-limb arteriopathy and limited availability of functional peripheral vascular access. It is commonly utilised in cases involving type 2 or 3 aortic arches necessitating right CAS, or in instances of type 2 bovine arches requiring left CAS. Access is accomplished by using a 21-G micro-puncture set. According to the RADCAR (RADial access for CARotid artery stenting) randomised controlled trial (RCT), the use of radial access for CAS resulted in 100% technical success, with low complication rates (access-site complications 0.9% vs. 0.8%; cerebral/heart events 0.9% vs. 0.8%). Nevertheless, the radiation doses reported were higher than with the transfemoral approach [25].

### Catheterization Technique

Catheterization of the common carotid artery (CCA) can be performed by using 5-Fr simple-angled catheters, catheters with second bends, or reverse-curve catheters, based on the aortic arch anatomy, with a 0.035″ hydrophilic wire, by using a left anterior oblique projection. For the catheterisation of the right CCA, right anterior oblique projection can be used to depict the origin and initial segment of the CCA. An arteriography of the ipsilateral intra-cranial arteries in Towne and lateral projection can be obtained prior to catheterisation of the internal carotid artery ICA.

Reverse-shaped catheters are proceeded over the wire until the ascending aorta. Subsequently, the guidewire is pulled back within the catheter and a manual twist of the proximal catheter will form the catheter within the ascending aorta; slowly pulling back whilst facing cranially will accomplish successful catheterisation of the main arch vessels.

A 90 cm 6-Fr working sheath should be substituted with the use of a 0.035″ wire or an alternative wire as needed – sometimes a stiffer wire is necessary. This wire should be advanced as deeply as feasible into the external carotid artery (ECA) and the sheath should be introduced up to 1 cm proximal to the carotid bifurcation, employing a method called the shuttle technique. The telescope technique represents an alternative, in which the sheath is advanced over the 4- or 5-Fr diagnostic catheter used for initial arch catheterisation and thus already placed in the common carotid artery to subsequently perform the catheterisation of the ICA. In general, this technique is faster, especially in straightforward type one great vessel origin. In more challenging anatomies, dedicated anchoring techniques are necessary to achieve a good outcome. A steep ipsilateral oblique projection usually distinguishes the carotid bifurcation.

Upon achieving an appropriate placement of the sheath, this is connected with a pressurised saline flush solution containing heparin at a ratio of 1 unit per mL of normal saline, which is delivered by a mechanical pump at a flow-rate of 300 mL per hour [26].

### Lesion Crossing

Using roadmap or other image-overlay techniques, a 0.014″ soft-tip crossing straight wire, shaped by the operator according to carotid anatomy and lesion characteristics, can be used to gently cross the ICA stenosis. An embolic protection device (EPD), if used, should be deployed distal to the stenosis. In some high-grade stenoses, short pre-dilation of the carotid stenosis with a 2–4 mm balloon may be required, to allow EPD or stent crossing, whilst it is also recommended to minimise bradycardia [27].

### Balloon Angioplasty

When considering pre-dilation, it is recommended to use balloons with diameters of less than 5 mm in order to reduce the risk of peri-procedural stroke or transient ischaemic attack [8]. Double dilation has been associated with statistically significant increases in neurological occurrences; thus, post-dilation should only be used if the completion angiography shows residual stenosis more than 30% [28]. Please note that the endpoint of CAS is not maximal luminal gain, but plaque coverage.

### Peri-procedural Medication

Within the context of the outlined procedural process, it is considered essential to administer peri-procedural heparinisation, with a suggested dosage ranging from 75 to 100 units per kilogramme, prior to crossing the lesion. The implementation of this strategy aims to achieve and maintain an activated clotting time (ACT) that lies within the optimal range of 250 to 350 seconds. The assessment of activated clotting time provides an important component in the procedural monitoring process, starting at the initial administration of heparin and continuing at 30-min intervals until the desired ACT range is consistently achieved throughout the duration of the procedure.

Furthermore, it is imperative to emphasise the administration of 0.6 to 1.2 mg of atropine prior to balloon inflation as a critical preventive measure against hypotension, bradycardia, or asystole [8].

If flow-limiting vasospasm is induced mechanically and still persists after the removal of the guidewire, 100-μg of nitroglycerin may be administered intra-arterially through the guiding sheath into the spastic carotid artery [27, 29].

### Stenting

After the stenosis has been located accurately using the roadmap technique, a self-expanding stent with a diameter 1 mm larger than the normal vessel diameter (e.g. 8mm in the CCA) and sufficient length to cover the stenosis (usually 40 mm) is deployed. The cranial edge of the stent should be preferentially deployed within a relatively straight ICA segment (to avoid excessive angulation that could incite thrombosis) and caudal edge within the distal CCA.

Completion angiography is imperative to confirm stent patency in two planes. This step is undertaken after the removal of the EPD, if one has been utilised. This assessment aims to rule out the existence of significant residual stenosis that might compromise blood flow and potentially lead to acute thrombosis, particularly in severely calcified and significant stenosis). Additionally, the angiography should verify the absence of a distal embolus and identify any signs of compromised flow in the end branches (in lateral and Towne projections).

### Embolic Protection Devices

Carotid artery stenting entails a risk of thrombotic particles dislodging from the plaque and migrating to the brain, causing a stroke. In order to mitigate this risk, embolic protection devices (EPDs) have been devised to capture and remove any fragments that may break off during the procedure. These devices are intended to create a barrier between embolic material and the brain, thereby decreasing the risk of stroke and improving the overall safety and efficacy of carotid artery stenting. It is important to note that the literature provides evidence supporting the increased likelihood of peri-operative stroke in the context of unprotected carotid stent placement [30, 31].

Types of Embolic Protection Devices:

1. **Distal filters** offer protection distal to the angioplasty site, and are positioned in the ICA before pre-dilation, to avoid the risk of embolization of atheromatous thrombi during manipulations. Precise placement in a downstream and straight segment of the ICA is important in order to prevent spasm and retrieval difficulties. In instances of tortuous or severely stenotic vessels, a ‘buddy-wire’ system may be employed, involving a 0.014-inch wire placed across the lesion to straighten the artery, thereby reducing tortuosity. In cases of challenging EPD passage, dilation of the stenotic segment using a 2 mm balloon may be necessary [32].
  After the CAS procedure, the EPD removal is undertaken using a retrieval catheter. In some instances, aspiration of thrombotic components from the EPD prior to retrieval might be necessary, which may be accomplished using a large lumen aspiration catheter that is positioned in contact with the EPD, and whilst applying suction using a 60ml syringe. Although rare, this complication may occur, leading to carotid artery occlusion and an accompanied acute stroke.
2. **Proximal embolic protection devices**, which include proximal balloon occlusion and flow reversal devices, serve the purpose of protecting the brain by reversing the stream of blood flow at the bifurcation, and allowing safe internal carotid artery stenting [30]. Flow reversal is accomplished through the selective obstructing of blood flow in the common carotid and external carotid arteries; thus, it is advisable to avoid placing proximal EPD in individuals who have significant atheromatous disease in those vessels [33]. This technology is ideal in highly tortuous or calcified internal carotid arteries, addressing potential challenges associated with the navigation and placement of distal EPD [30].

The optimal brain protection system during CAS continues to be a matter of discussion, as the existing research presents varying and inconclusive findings. Proximal protection utilising flow reversal has been linked to diffusion-weighted imaging (DWI) lesions, however to a lesser extent when compared to distal EPDs [21, 34].

### Advanced Techniques

#### Transcarotid Percutaneous Access

Access can be gained directly to the proximal CCA, via direct puncture. An arterial sheath is used in addition to a carotid artery flow-reversal EPD. This technique may be of benefit in patients with aortic arch disease, as it eliminates the need for catheter manipulation within the arch. Relative contraindications to this technique include lesions that are ≤ 5 cm distal from the clavicle; serious vascular tortuosity or calcification; and small diameter of the common carotid artery [3].

#### Multi-level Lesion Treatment

Lesions in the brachiocephalic trunk or proximal CCA in conjunction with severe atheromatous disease in the ipsilateral ICA are known as ‘tandem disease’ and are treated with a hybrid procedure: retrograde endovascular angioplasty at the level of the brachiocephalic trunk/CCA, which is then followed by CEA of the ICA. In a study of 700 procedures, the reported mortality/stroke rate was 3.3% at 30 days and remained the same after a median follow-up of six years [8].

Tandem lesions entail intracranial large-vessel occlusion and concurrent stenosis of the cervical internal carotid artery and these lesions can be either chronic or present as an acute on chronic occlusive event, usually of the proximal or distal ICA (carotid terminal ‘T’ occlusions). The performance of emergency carotid stenting in the acute setting of intracranial mechanical thrombectomy (MT) is beyond the scope of this document. In general, CAS can be executed during an intracranial MT procedure, or not at all, with the specific approach being contingent upon the clinical and technical characteristics of the case [35, 36].

## Post-Procedural Care, Medication and Follow-Up

Patients should be admitted for at least 24 h surveillance and it is not advised to consider performing CAS as a day-case procedure, due to the risk of severe complications that may require immediate treatment. Post-procedural care should include:

- Routine evaluation of neurologic function and access-site.
- Serial monitoring of the vital signs in a specialised care unit [12]. It is imperative to carefully monitor and regulate the blood pressure, ensuring that the systolic pressure remains below 140 mmHg and the diastolic pressure remains below 90 mmHg, in order to prevent the occurrence of hyperperfusion syndrome. Bradycardia and/or hypotension could occur following balloon dilation or irritation due to manipulations at the carotid bulb due to the activation of baroreceptors, and fluid resuscitation is usually adequate. If haemodynamic lability presents despite prophylactic IV atropine administration (0.5 – 1.0 mg) the use of inotropes IV administration protocols is advised [37, 38].
- In cases of compromised renal function prior to the procedure, monitoring and hydration based on the European Society of Urogenital Radiology (ESUR) guidelines should be performed, as per peripheral arterial interventions [36, 39].

Daily dual antiplatelet therapy consisting of 75–100 mg of aspirin and 75 mg of clopidogrel is required. Aspirin monotherapy at a dosage of 75–100 mg orally once daily should be continued indefinitely following one month [8, 40]. Nevertheless, prolonged > 6 months dual antiplatelet therapy in CAS, especially following the use of dual-layer stents, is still under investigation [41].

Low-dose statins should be commenced if the patient is not already on statin therapy.

Baseline Doppler ultrasound examination prior to discharge is extremely useful for future accurate in-stent restenosis detection. Standard follow-up at 1 and 6 months and yearly thereafter is advised and should include clinical and DUS examination for in-stent restenosis assessment. DUS of the contralateral ICA is advised during pre-scheduled follow-up visits. Termination of surveillance should be considered only in patients that have been deemed unfit for further interventions.

## Outcomes

### Symptomatic and Asymptomatic Patients

Over the past decades, several randomised controlled trials and their meta-analyses have compared CAS with CEA. Despite variability in selection criteria and study endpoints, outcomes consistently indicated an increased peri-procedural risk of stroke following CAS, and a higher risk of myocardial infarction and cranial nerve injury following CEA [42]. In 2021, the European Stroke Organisation (ESO) issued recommendations on treatment of patients with symptomatic or asymptomatic atherosclerotic carotid stenosis, supported by systematic review and meta-analysis of RCTs [2].

#### Symptomatic Patients

The 2021 ESO meta-analysis included a total 7 randomised control trials (RCTs) with 4,893 patients comparing short-term risks and long-term effects of CAS (transfemoral access only; no RCTs for transcarotid or transradial CAS) versus CEA, concluding that there is moderate-quality evidence indicating the superiority of CEA, driven mainly by the greater peri-procedural stroke rates following CAS. On the other hand, CEA was again correlated with higher risk of myocardial infarction and cranial nerve palsy (mainly transient). However, a high level of evidence indicated a higher risk of peri-procedural stroke or death in patients > 70 years for CAS (low-quality evidence in patients < 70 years). Additionally, the risk of peri-procedural stroke/death was again higher when CAS was performed within the first week following the ischaemic event. Nevertheless, CAS was equally effective to CEA in preventing long-term stroke (moderate grade evidence).

The timing of CAS after stroke has been also investigated. According to a secondary analysis of the German Statutory Quality Assurance Database, an increased risk of new stroke and mortality was associated with a short time interval between the neurologic index event and CAS of up to 7 days [42]. Meanwhile, a new 2022 meta-analysis reported a higher risk of death at < 2 days than 3–14 days, and the same risk at 7 days as at 8–14 days and a higher 30-day stroke incidence if CAS was performed at 7 days versus 8–14 days [43].

#### Asymptomatic Patients

Data comparing the short-term risks and long-term effects between stenting and endarterectomy for asymptomatic carotid stenosis were available from seven trials including a total of 3,373 patients. Overall, there was no clear evidence of statistically significant differences in outcomes between endarterectomy or stenting (low- to moderate-quality evidence) [2].

Only recently, more data were made available regarding asymptomatic patients, which were not included in the ESO guidelines document, and could change future guidelines in favour of CAS.

The second asymptomatic carotid surgery trial (ACST-2) was a multi-centre RCT, conducted in 130 centres, which randomised a total of 3,638 patients with asymptomatic carotid stenosis to CAS (n = 1,811) versus CEA (n = 1,814). The procedural disabling and not-disabling stroke rates were 1% and 2% respectively and according to Kaplan–Meier estimation, the 5-year rates of non-procedural disabling and any stroke were similarly low for both groups (2.5% in each group, and 5.3% CAS versus 4.5% CEA, respectively; p = 0.33). Following each revascularisation method, the annual rate of disabling or fatal stroke was approx. 0.5%. There were no statistically significant differences in procedural death or any fatal or disabling stroke, procedural death, or any stroke, non-procedural fatal or disabling stroke, and non-procedural stoke. The authors also performed an additional meta-analysis of all CAS versus CEA trials, and concluded that non-procedural stroke risk was similar between symptomatic and asymptomatic patients (RR 1.11, 95% CI 0.91–1.32; p = 0.21) [2].

More recently, in a study involving a large patient-level data analysis, 2,500 asymptomatic, non-octogenarian subjects, were treated with CAS versus CEA [37]. Again, CAS performed equally to CEA with regards to the composite primary endpoint of peri-procedural stroke, myocardial infarction or death during the period, or ipsilateral stroke within four years (5.3% vs 5.1%; respectively; HR 1.02; 95%; CI 0.7–1.5). Moreover, similar results were noted for any stroke (2.7% CAS vs. 1.5% CEA), death (0.1% CAS vs. 0.2% CEA) and any stroke or death (2.7% CAS vs. 1.6% CEA). MI was significantly higher following CEA (0.6% CAS vs. 1.7% CEA; p = 0.01). Ipsilateral stroke rates were similar after the peri-procedural period (2.3% CAS versus 2.2% CEA) [37].

Finally, according to the national German 2014–2019 registry, the in-hospital risk of disabling stroke or death for asymptomatic patients undergoing CAS or CEA during was 0.7% (indicating an estimated 30-day risk of approx. 1%) for each procedure [44]. The ongoing CREST-2 multi-centre RCT will provide more data regarding the choice of CAS versus best medical treatment alone [45].

A systematic review of 46 studies with 5,018 patients noted higher age, plaque vulnerability and complex carotid-aortic arch anatomy as risk factors for the occurrence of new ischaemic brain lesions [46].

#### Stent Technologies

The use of open-cell stents (free-cell area > 5 mm) or closed-cell stents has also been investigated, although the evidence remains limited. Both technologies demonstrate advantages and disadvantages. In a pooled analysis of individual patient data from the International Carotid Stenting Study [47], EVA-3S [48], and SPACE trials [49], the risk of procedural stroke or death in symptomatic patients was lower in the CCS group (6.0%) vs. the OCS group 10.3%) (RR 1.76; 95% CI 1.23–2.52; p = 0.002) [47]. In another meta-analysis of 9 studies including 8,018 patients who underwent 8,028 CAS procedures (4,018 open-cell stents; 4,010 closed-cell stents), nearly half of the patients (3,452, 43.1%) were symptomatic, with no significant difference between the closed- and open-cell stent groups. During the first month after the procedure, there were no significant differences in mortality (OR 0.69, 95% CI 0.39 to 1.24), transient ischaemic attacks (OR 0.95, 95% CI 0.69 to 1.30, p = 0.74), or strokes (OR 1.17, 95% CI 0.83 to 1.66) [50]. The International Carotid Stenting Study of 825 patients also concluded that there was no significant difference in the risk of severe restenosis (≥ 70%) after open-cell stenting (n = 27) versus closed-cell stenting (n = 43; 5-year risks, 8.6% versus 12.7%; unadjusted hazard ratio, 0.63; 95% CI, 0.37–1.05), and that risk of ipsilateral stroke beyond 30 days after treatment was similar with open-cell and closed-cell stents (hazard ratio, 0.78; 95% CI, 0.35–1.75) [51].

Moreover, in a small study including 52 patients undergoing OCS deployment in one artery and CCS in the contralateral artery, stent selection was based on arterial anatomy and lesion morphology; This study demonstrated similar peri-procedural cerebral ischaemic events, long-term stent patency, and stroke recurrence between the two groups, allowing the authors to conclude that optimal stent selection could be individualised [52].

In a 2021 retrospective analysis of 2,671 CAS procedures with distal embolic protection in the Society for Vascular Surgery Vascular Quality Initiative (VQI) database from 2016 to 2018 (51.8% CCS and 48.2% OCS), CCS use was correlated with an increased odds of in-hospital stroke/death for carotid bifurcation lesions [53]. A similar meta-analysis of two randomised controlled trials and 66 cohort studies indicated that stent design is not associated with short- or intermediate-term clinical major adverse event rates in patients undergoing CAS, but that use of OCS predisposed to a 25% higher chance (RR, 1.25; P 1/4 0.03) of developing postprocedural new ischaemic lesions on MR-DWI [54].

More recently, micromesh stent technology has developed to address the disadvantages of both OCC and CCS (flexibility with reduced free-cell area). However, available data remain very limited and only small studies have been published, indicating optimistic results [55–57]. Short- and long-term safety and efficacy of a dual-layer micromesh stent (DLMS) has already been shown in a number of studies [21, 58, 59]. A current multi-national, multi-specialty study aims to expand the clinical evidence base and confirm the good performance of the DLMS, now in a large, unselected patient cohort eligible for an elective CAS treatment as per routine care. Simultaneously, the study aspires to provide valuable insights into the contemporary pan-European clinical CAS practice trends. The design of the Roadsaver study has been described earlier [21]. Two meta-analyses evaluated the 30-day clinical safety of the currently available DLMS (Roadsaver™ and CGuard™) in a pooled dataset concluding that the two DLMS do show promising safety profiles, but there is no substantial evidence to recommend a specific device [60, 61].

#### Protection Devices

Currently, there is a Class IIa Level of Evidence C recommendation of the joint ESC/ESVS 2017 guidelines regarding the use of embolic protection devices during CAS [8]. However, the level of evidence remains low, whilst there is no evidence for the benefit of a specific EPD versus another. The use of new EPD such as dynamic flow reversal during transcarotid CAS seem to provide satisfactory neuroprotection as very low ipsilateral stroke rates (0.6% at one-year follow-up) have been reported [62].

#### Alternative Access Outcomes

Whilst the transfemoral approach has traditionally been considered the gold-standard access for CAS, there is an increase in popularity towards alternative accesses. These alternatives, particularly radial artery access, are being considered more frequently, primarily due to the significant advantage of a reduced risk of puncture site bleeding. Various authors have reported very optimistic results from alternative access options such as transcervical and radial artery access. Transcervical access using surgical cut down to access the CCA has the clear advantage of avoiding arch manipulations that could lead to thromboembolic events, especially in difficult arch anatomy [63]. In a 2013 systematic review, the authors analysed outcomes of 12 studies, which included 722 mixed symptomatic and asymptomatic patients, who underwent 739 transcervical CAS procedures (filter-protected, under reversed flow and unprotected). Technical success was 96.3% with an incidence of open conversion of 3.0%. Access-related complications were 2.9% (17/579 procedures), and included 15 haematomas and 2 transient laryngeal palsy cases. Two fatal strokes were noted. The overall rate of stroke was 1.2% (3/250) in direct CAS with transcervical access and 1.02% (5/89) in CAS under reversed flow (non-significantly different). TIA rate was 2.7%. The rates of stroke, myocardial infarction, and death was 1.1%, 0.14%, and 0.41%, respectively [64].

Percutaneous, direct, transcarotid access (CCA access) has been proposed as a safe alternative to transfemoral access for CAS. Bergeron et al. describe the technical details of ultrasound-guided percutaneous transcarotid access and reported their experience in 52 patients. The authors reported 100% technical success, no access-related complications, and no death, stroke, TIA, nerve palsy, or cardiac events [65].

The transradial approach has also been reported to be safe and effective. In 2022, the outcomes of the multicentre prospective CREST-2 registry on transradial approach were published. Amongst 2,868 cases undergoing CAS for asymptomatic disease, transradial access was performed in 213 patients (3.9%). Similar outcomes were noted for the composite endpoints of major access-related complications (0% transradial vs. 1.1% transfemoral) and peri-procedural stroke or death (3.3% transradial vs. 2.4% transfemoral; OR = 1.4 95%CI 0.6, 3.1; p = 0.42) [65].

## Complications

In general, rates of access-related complications (transfemoral, transradial) and procedure-related acute kidney injuries should not differ from those reported following peripheral arterial procedures, although comparative studies are missing from the literature. Regarding the CAS-related major complications, the ESO expert consensus recommended that the independently assessed risk of in-hospital stroke or death following CAS for symptomatic carotid stenosis should not exceed 4% and 2% for asymptomatic carotid stenosis. The 30-day rates should not exceed 3% and 6% for asymptomatic and symptomatic patients, respectively [2].

The expected immediate-early complications include:

o embolic events leading to TIA or stroke;
o acute in-stent thrombosis that usually leads to major stroke;
o reperfusion injury;
o retroperitoneal bleeding;
o expanding groin haematoma;
o pseudoaneurysm;
o acute kidney injury; and
o haemodynamic lability following balloon dilation.

## Conclusion

Carotid artery stenting is a minimally invasive and efficacious approach for the management of carotid artery disease, a primary contributor to the incidence of stroke.

Based on extensive review of existing evidence and established guidelines, the SOP writing group has identified the key recommendations for carotid artery stenting:

*Patient selection:* Carotid artery stenting is indicated for patients presenting with symptomatic high-grade carotid artery stenosis for a variety of reasons, and in certain instances of asymptomatic stenoses.

*Procedural technique:* The technique refers to a systematic and structured approach that is used to accomplish a specific task or objective. It involves a series of steps or actions that are taken in a particular order so that the task is completed efficiently and effectively, with minimal errors or deviations from the intended process. It is recommended that the procedure be carried out by trained operators who have received adequate training in the field of carotid artery stenting. The utilisation of embolic protection devices is advised as a means of mitigating the likelihood of stroke incidence during the intervention.

It should be noted that the objective of CAS endpoint is not to achieve maximal luminal gain, but rather to attain complete plaque coverage.

*Post-treatment care:* Immediate post-procedural monitoring and long-term follow-up is recommended in order to observe and assess for potential complications.

It is recommended that patients be provided with continuous medical management in order to reduce the risk of future cerebrovascular events.

## Abbreviations

ACST-2: Second Asymptomatic Carotid Surgery Trial
ACT: Activated clotting time
CAS: Carotid artery stenting
CCA: Common carotid artery
CCS: Closed-cell stents
CEA: Carotid endarterectomy
CFA: Common femoral artery
CI: Confidence interval
CREST-2: Carotid Revascularisation and Medical Management for Asymptomatic Carotid Stenosis Trial
CT: Computed tomography
CTA: CT angiography
DAPT: Dual antiplatelet therapy
DLMS: Dual-layer micromesh stent
DSA: Digital subtraction angiography
DUS: Doppler US
DWI: Diffusion-weighted imaging
ECA: External carotid artery
ECST: European Carotid Surgery Trial
EPD: Embolic protection device
ESO: European Stroke Organisation
ESUR: European Society of Urogenital Radiology
ESVS: European Society for Vascular Surgery
HR: Hazard ratio
ICA: Internal carotid artery
IV: Intravenous
MRA: Magnetic resonance angiography
MR-DWI: Magnetic resonance diffusion-weighted imaging
MRI: Magnetic resonance imaging
MT: Mechanical thrombectomy
NASCET: North American Symptomatic Carotid Endarterectomy Trial
OCS: Open-cell stents
RADCAR: RADial access for CARotid artery stenting
RCT: Randomised controlled trial
RR: Relative risk
SRU: Society of Radiologists in Ultrasound
TIA: Transient ischaemic attacks
US: Ultrasound
VQI: Society for Vascular Surgery Vascular Quality Initiative
